# Vision Marker-Based In Situ Examination of Bacterial Growth in Liquid Culture Media

**DOI:** 10.3390/s16122179

**Published:** 2016-12-18

**Authors:** Kyukwang Kim, Duckyu Choi, Hwijoon Lim, Hyeongkeun Kim, Jessie S. Jeon

**Affiliations:** 1 Urban Robotics Laboratory, Korea Advanced Institute of Science and Technology, 291 Daehak-ro, Daejeon 34141, Korea; kkim0214@kaist.ac.kr; 2 Department of Mechanical Engineering, Korea Advanced Institute of Science and Technology, 291 Daehak-ro, Daejeon 34141, Korea; duckyu@kaist.ac.kr (D.C.); hkkim1227@kaist.ac.kr (H.K.); 3 School of Electrical Engineering, Korea Advanced Institute of Science and Technology, 291 Daehak-ro, Daejeon 34141, Korea; wjuni@kaist.ac.kr

**Keywords:** bacterial growth, vision marker, fast Fourier transformation, microfluidics

## Abstract

The detection of bacterial growth in liquid media is an essential process in determining antibiotic susceptibility or the level of bacterial presence for clinical or research purposes. We have developed a system, which enables simplified and automated detection using a camera and a striped pattern marker. The quantification of bacterial growth is possible as the bacterial growth in the culturing vessel blurs the marker image, which is placed on the back of the vessel, and the blurring results in a decrease in the high-frequency spectrum region of the marker image. The experiment results show that the FFT (fast Fourier transform)-based growth detection method is robust to the variations in the type of bacterial carrier and vessels ranging from the culture tubes to the microfluidic devices. Moreover, the automated incubator and image acquisition system are developed to be used as a comprehensive in situ detection system. We expect that this result can be applied in the automation of biological experiments, such as the Antibiotics Susceptibility Test or toxicity measurement. Furthermore, the simple framework of the proposed growth measurement method may be further utilized as an effective and convenient method for building point-of-care devices for developing countries.

## 1. Introduction

A bacterial culture is one of the most basic and core experimental method used for various applications in research, clinical, and industrial fields. Many commercially available automated systems for bacterial growth measurements using the optical density (OD) of the liquid broth were developed, and are currently used as a de facto standard for the growth measurement. Many frequently performed experiment protocols, such as plasmid transformation, require growth of bacteria until the OD value of the broth reaches a certain value. However, most of these OD measuring devices are very costly which can be inappropriate for the clinics or laboratories in developing countries where bacterial infection outbreaks are frequent. In addition, these sensitive devices are not appropriate for on-site operation where a low-cost growth detection system might be required. These devices can cause issues in maintenance as the current systems also require intensive maintenance procedures, including calibration and alignment of the light sensors.

The cost problem can be solved as a low-cost 3D-printable colorimeter, based on the OD, was proposed [1]. Nonetheless, non-automatized OD measurement devices require manual filling of broth to the cuvette, thus making it difficult to render the biological process automated and they are also labor-intensive. Moreover, this 3D-printable OD device requires alignment of the LED and light sensor to measure OD properly. The manual culture process, relying on the human eye and decision, is rather cheap but it is very labor intensive [2] and requires training of the technologists.

In this study, we propose a simple and robust system, which does not require as accurate system setup nor management compared to the conventional methods, to measure the visibility of the bacteria-growing liquid broth by using a marker and a camera. We focused on the fact that if the image is blurred, the fast Fourier transform (FFT) spectrum pattern of the blurred image has losses in the high-frequency region compared to the pattern of the original image, thus decreasing the spectrum pattern size [3]. It is expected that the increase of bacterial cells in the culture broth blurs the image of the object (marker) placed on the backside of the culture tube. The principle of the new system is very similar to the principle of the OD measurement, except that the new system is much simpler, as it only requires close enough distance to take a clear photograph. The experiments to check the proposed idea showed that the image processing could clearly distinguish the blurring of the broth by using the FFT and the marker.

Recent research showed that the culture of bacteria under a well-controlled microenvironment shows a higher growth rate compared to a classical 1 L batch culture [4], demonstrating that the polydimethylsiloxane (PDMS) Lab-on-a-Chip devices are also a candidate platform for the bacterial culture. To increase the density for efficiency and to test whether this method works at milli/microfluidic scale, a simple PDMS device with a visible marker for bacteria culture and FFT-based growth detection was developed.

## 2. Materials and Methods

### 2.1. Platform Overview

**Figure 1:**
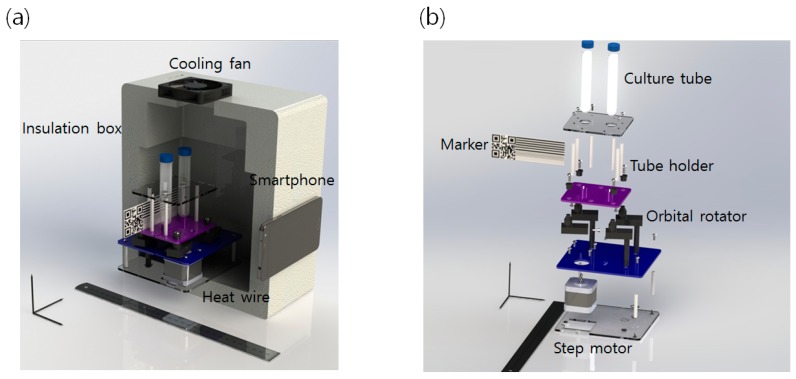
3D rendering image of the developed automated shaking incubator. (**a**) Cross-sectional diagram and (**b**) exploded diagram. The ruler was added for scale comparison.

The electronically controlled shaking incubator was assembled for automated image acquisition as shown in Figure 1. The automation was made possible as the inner rotator temporally stopped to acquire an image of the culture bottles at every examination time. Each mechanical part was made using a 3D printer, and a Styrofoam box with a household heating wire was used as a heating source. The images of the culture bottle are acquired with a smartphone camera via an Android application developed for this purpose. For the data acquisition, the electronics system of the shaking incubators was designed to stop shaking for 1 min per every 1 h.

### 2.2. Bacterial Culture and Experiment Settings

The two bacterial strains (*Escherichia coli* DH5-α and *Pseudomonas aeruginosa*) were selected for the experiments. Measuring the growth of these species is important as the *E. coli* is one of the main causes of the bacteremia (about 45% of the gram-negative bacteria detected from septicemia patients are *E. coli*) [5] and *P. aeruginosa* is also a major gram-positive blood-isolated bacteria from the septicemia patients. Measuring the growth state of the *E. coli* is also used for the toxicity measurement experiment.

The bacterial strains were kindly supported by Molecular Physiology Laboratory and Nanobiomedicine Laboratory of KAIST. The stock was thawed and pre-cultured in the liquid broth media overnight. A platinum loop was used to inoculate the bacteria into the medium, which is installed to the FFT measurement device. The *E. coli* was cultured with the Luria-Bertani (LB) broth in the assembled incubator at 37 °C and at a shaking speed of 120 rpm (rotation per minute). *P. aeruginosa* was cultured under the same condition in Tryptic Soy Broth (TSB).

### 2.3. Image Processing Algorithm

A horizontal striped pattern marker was used for the measurement of bacterial growth in the culture bottle. The marker was placed at the backside of the culture bottle so that the camera could scan the marker through the liquid broth. When the marker in the image is blurred, the high-frequency domain of the image decreases. A 2D spectrum image was obtained by the FFT, and the area of white regions in the spectrum image is calculated after thresholding to reduce the noise (threshold value of 127 used). A horizontal marker generates “|” shaped long white spectrum patterns and the area/length of this pattern is shortened as the bacteria grow.

**Figure 2:**
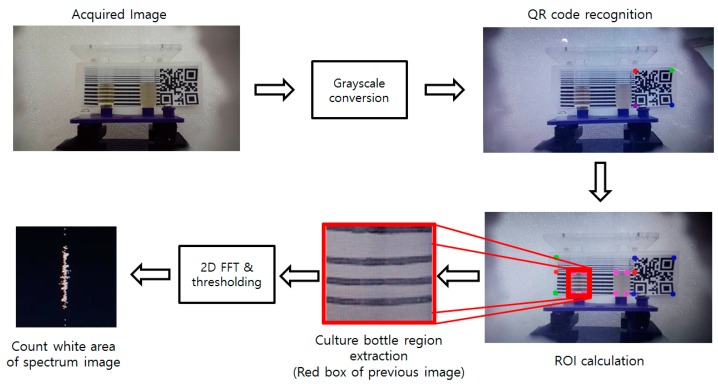
Flow chart describing the proposed image processing algorithm. Dots in the images indicate the recognized corners of the QR code and striped regions.

The quick response (QR) code was used to obtain the position of the rotator, thus defining the region of interest (ROI) for the image processing. The striped region of the marker was designed to be double the length of the QR code’s length, and the location of the culture bottle is fixed, making it easy to obtain an accurate ROI from the image. Overall algorithm is shown in Figure 2.

### 2.4. Preparation of Millifluidic Culture Environment

**Figure 3:**
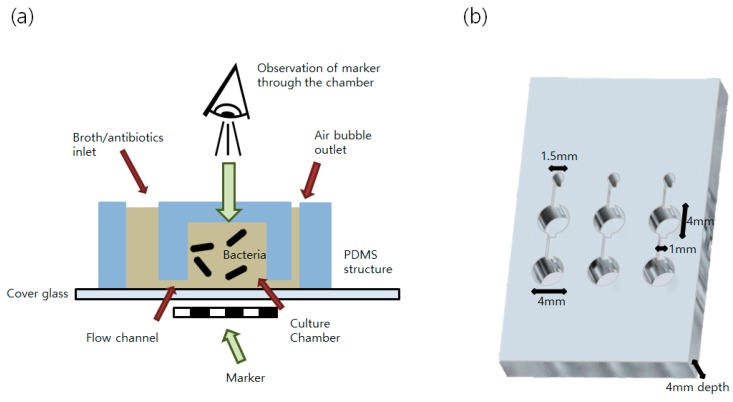
Schematic drawing of the designed polydimethylsiloxane (PDMS) devices. Cross-sectional diagram (**a**) and 3D view (**b**) of the designed PDMS device for observing bacterial growth is shown.

We also extend the new measuring system using the PDMS devices. For bacterial culture and broth injection (containing antibiotics or toxicity testing solution), a single-chambered structure inspired by Choi et al. [6] with a large inlet and small air bubble outlet were designed as shown in Figure 3. The flow channel (1 mm × 1 mm × 1 mm) connects the inlet to the culture chamber and the chamber to the air bubble outlet. The inlet was used for the injection of broth and antibiotics/toxic materials to the culture chamber, and the outlets were used to remove bubbles blocking the sight of the culture chamber for the growth examination.

The device was fabricated by using a modified protocol proposed by Shin et al. [7]. Stereolithography (SLA) 3D printer (Nobel 1.0 printer of XYZprinting, Inc., San Diego, CA, USA) fabricated molds were used instead of the silicon wafers for curing PDMS devices. The ratio of the silicone elastomer base and the curing agent was increased to 12:1 for higher adhesion force to a relatively rough surface generated by the 3D printed mold.

## 3. Results and Discussion

### 3.1. Detection of Bacterial Growth in Liquid Broth

**Figure 4:**
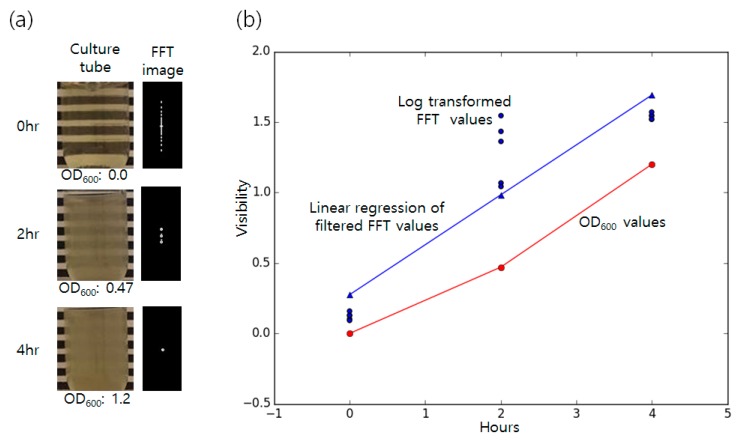
Visibility of the bacteria at different time points. (**a**) Visibility of the culture bottle (left) and fast Fourier transform (FFT) pattern (right) changes as time passes (0, 2, 4 h). The corresponding conventional OD_600_ values are shown for each time point; (**b**) The plot shows the change of the measured visibility as time passes. The red line indicates the OD_600_ value of the sample at a given time. Blue dots are log transformed visibility obtained by the FFT measurement (ratio of the blurred region count and blank region count). Count means the size of the white region of the FFT images. The blue triangles and line show linear regression result of the FFT measured values. The OD_600_ and the FFT result showed a correlation coefficient of 0.992.

The shaking incubation made bacteria grow rapidly and saturated the visibility of the culture bottle after 4 h of incubation. Five images were obtained per every 2 h (Images from 0, 2, and 4 h were gathered). Comparison of the clear culture tube and the inoculated tube was done by dividing the spectrum area of the blurred inoculated bottle by the spectrum size of blank bottle to check the difference in the ratio, as shown in Figure 4.

As the bacteria grew in the culture tube, the spectrum area of the FFT image decreased, making the ratio between two bottles decrease rapidly and significantly as time passed from 0.76 ± 0.018 (0 h) to 0.03 ± 0.0006 (4 h) (one-way ANOVA, *p*-value < 0.01). It took about 4 h to detect the growth of bacteremia, showing no huge difference with the commercially available systems that normally take 4 to 5 h.

For the comparison with the traditional technique, OD_600_ (OD at the 600 nm wavelength) values were also measured by taking 1 mL of culture broth in the cuvette for the measurement. (Ultrospec 7000, GE, Boston, MA, USA.) The clear culture broth was used as a control. The 0 h sample showed OD of 0.0 while it increased to 0.47 after 2 h of incubation and reached a value of 1.2 by the saturation point of 4 h. For the comparison of the OD_600_ values and the FFT values, the FFT were log transformed using the following absorbance calculation formula,

(1) $$A=-{\text{log}}_{10}(T_{i}/T_{t})$$

where *T_i_* is a received radiant flux and *T_t_* is a transmitted radiant flux in OD calculation. As radiant flux is not available in the case of the FFT values, measured spectrum area count of the sample was used as the *T_i_* value and the area value of the control (clear broth) was used as the *T_t_* value. The result plotting showed well centered data at the full growth state (4 h) and no growth state (0 h), but some outliers were found at the interval time (2 h). In order to filter out the obvious outliers due to experimental error, when the FFT value of the control tube is too low (lower than the mean value of the first five samples at time 0), the value was discarded (removing too small *T_t_*). The median value from the dataset with five samples per each measurement hour were computed and used for the linear regression. The median was used to reduce errors caused by unexpected small/large *T_i_* value inputs. The final formula *y* = 0.35*x* + 0.206 was obtained as a regression result after the filtering. Then, the Pearson correlation coefficient of the OD_600_ values and the FFT linear regression result was calculated to check the linear correlation of two methods. The correlation coefficient *r*^2^ value of 0.992 was obtained which shows that the FFT calculation results have good correlation with the OD measurement.

Compared to the conventional techniques, the combination of the camera and marker is cheaper and simpler as fewer adjustments and precision are required for the operation. Only the striped pattern image at the backside of the culture tube is required for the growth examination; no fixed camera settings or liquid vessels are required, except the clearly focused image for right FFT output. The conventional OD measurement task requires a sample of filling the bacteria growing broth to a cuvette with fixed size. This makes it difficult to render these experiments automated, and they are also labor intensive. The proposed vision-based system does not require a manual checkup, as it is directly linked to the camera in the incubator. Also, because the marker is non-invasive, the proposed system has less possibility of contamination during the measurements than the conventional methods. Although the OD cannot be fully replaced, as the OD has higher resolution (OD is log scale), the FFT-based method can replace the OD in growth/no-growth tests. Simplified procedures allow an increase of throughput by taking a picture of multiple samples at once, or possibly aiding automation of other biological experiments requiring the examination of bacterial growth. Table 1 was added to summarize the differences between the OD measurement and the proposed method.

### 3.2. Expansion of the Concept to the Lab on a Chip Device

**Figure 5:**
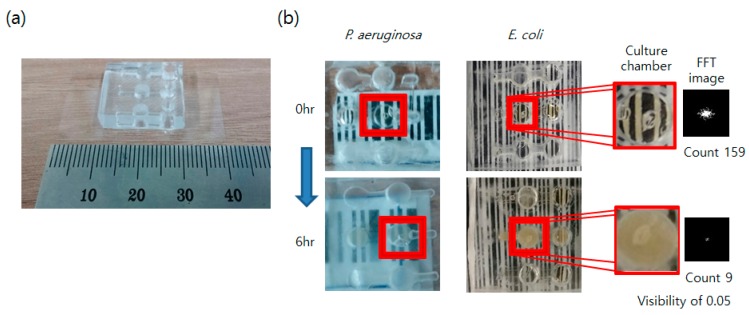
Usage of PDMS devices for bacterial growth. (**a**) PDMS device is fabricated and used for the bacterial culture (Imaged with 40 mm marked ruler). (**b**) The growth of *P. aeruginosa* (left) and *E. coli* (right) in the culture chamber of the PDMS device is shown. The FFT spectrum of the red-squared region (culture chamber on the marker) shows a decrease in the high-frequency region as time passes due to bacterial growth.

To increase screening efficiency and test the proposed concept at the various scales, we developed a simple PDMS device with a marker under the growth chamber for the bacteria culture. The results showing bacterial culture in the PDMS chip are shown in Figure 5.

The experiment using the PDMS devices showed similar results compared to the macro-scale culture and detection; the striped pattern marker placed under the culture chamber blurred after 6 h of incubation. The clear striped pattern, which was observable through the culture chamber at the start of incubation (0 h), almost disappeared after 6 h. The FFT pattern area of the culture chamber was 159 when the broth was first injected (0 h), but the value decreased to 9 at 6 h. This value resulted in the normalized visibility value being 0.05, similar to the result shown in Figure 4. This shows that the principle of the blurred marker can also be used at various scale systems, including milli/microfluidics. As shown in Figure 5, both experiments with the *E. coli* and *P. aeruginosa* showed similar results in growth time, broth blurring state, and FFT pattern. Because the PDMS device is not optimized for the continuous culture of the bacteria, it took about an additional 1~2 h to reach the saturated growth state. Adding more structures for promoting the growth, such as aeration without evaporating liquid broth, would possibly allow a faster performance.

The Antibiotics Susceptibility Test (AST) or toxicity measurement experiments are generally performed in various concentration conditions and multiple experiment sets. We expect that using the PDMS devices with multiple culture chambers can aid to increase the throughput of the experiment in these cases. Many other biological experiment protocols or environmental applications require the growth of bacteria. The developed PDMS devices with the proposed growth detection method may also be used for the detection of coliforms, where conventionally, the chromogenic enzyme substrate assay is used for checking whether the bacteria grow in the coliform in multiple samples.

Although recently, SLA 3D printers are becoming cheaper than before, Fused Deposition Modeling (FDM) type printers are still more widely used as they are distributed more and easier to access. However, FDM printed objects have a rough surface as grains are formed due to the deposition of plastic filaments. These grains cause chinks during the PDMS templating and interrupt the bonding of molded PDMS to the cover glass. The attempts to remove the grains using acetone vapor were not effective as the melted surfaces were rougher compared to the SLA printed outputs. While using SLA type printers for fabricating the templates for PDMS molding is recommended, finding ways of using the FDM printers for the printing master template will be useful as they are more cost effective.

We expect that the proposed method can be used in various applications where automatic examination of bacterial culture would be beneficial or necessary. One potential application is the AST. The survival rate of the bacteremia or septicemia patient decreases by 9% for every passed hour [8]; the detection of bacterial growth when certain antibiotics are given is a crucial task. As mentioned previously, bacterial infections occur more at the regions where access to well established social infrastructures are limited. Also, the AST requires three sets of experiments per patient [8]. Automation of these processes with the proposed method can help to decrease the required labors and aid doctors to make consistent decision by reducing the fatigue.

Another biological experiment for environmental applications also requires the growth examination of the bacteria. Co-culture of the *E. coli* and testing chemicals, such as heavy metal ions, is used for the examination of the toxicity of the given chemical substances [9,10]. The chromogenic enzyme substrate assay, which cultures the water sample with the broth containing X-gal (also abbreviated as BCIG for 5-bromo-4-chloro-3-indolyl-β-d-galactopyranoside) is used for checking whether the coliform exists in multiple samples [11]. These experiments are considered to be simple but are disregarded due to the lack of an easy and automated method to check bacterial growth with the electronics system, making the protocol labor intensive. Further extension of the proposed method to the PDMS devices’ scale could be useful in the automation of PDMS-based culture methods or in bringing other large-scale experiments into the Lab-on-a-Chips.

## 4. Conclusions and Future Works

We have shown that the proposed method of FFT-based detection can be used to measure the growth of the bacteria in the culture tube and the designed PDMS device. The developed system is simpler and cheaper than the conventional OD measurement system, and therefore, the new system allows various applications and automation of various biological experiments. Although this method is not as accurate nor does it have as high resolution as classical OD sensors, the benefits such as low cost, high durability, and high replicability could be attractive for laboratories in developing countries. The automated 3D printable shaking incubator and the software for the continuous image acquisition were also developed and available online.

As a future work, we will focus on acquiring a higher resolution of the growth state and comparing OD values and FFT spectrum size ratio of standard culture tubes. The current system can easily distinguish growth/no-growth in various vessels, but some protocols require the growth state of certain OD values to proceed. Acquiring a higher resolution could help to apply this method to automatize more applications and protocols, and thus decrease the costs and lead to fewer facilities being required.

## Figures and Tables

**Table 1:** Summarized differences between OD- and FFT-based growth detection.

	OD	FFT
Cost	High	Low
Maintenance	Hard	Easy
Setup	Precise	Rough
Automation	Hard	Easy
Resolution	Standard	Lower
Accuracy	Standard	Bit Lower
